# Is vigorous-intensity physical activity required for improving bone mass in adolescence? Findings from a Brazilian birth cohort

**DOI:** 10.1007/s00198-019-04862-6

**Published:** 2019-02-22

**Authors:** R. M. Bielemann, V. V. Ramires, F. C. Wehrmeister, H. Gonçalves, M. C. F. Assunção, U. Ekelund, B. L. Horta

**Affiliations:** 1 Post-Graduate Program in Epidemiology, Federal University of Pelotas, Pelotas, Brazil; 2 Department of Nutrition, Federal University of Pelotas, Pelotas, Brazil; 3 Programa de Pós-graduação em Epidemiologia, Rua Marechal Deodoro, 1160–3° andar, Pelotas, Rio Grande do Sul CEP: 96020-220 Brasil; 4 Medical Research Council, Epidemiology Unit, University of Cambridge, Cambridge, UK; 5 Department of Sport Medicine, Norwegian School of Sport Sciences, Oslo, Norway

**Keywords:** Adolescence, Bone mineral density, Cohort studies, Vigorous physical activity

## Abstract

**Summary:**

The association between moderate and vigorous physical activity throughout adolescence and areal bone density (aBMD) at 18 years of age was evaluated. Vigorous-intensity physical activity at 11, 15, and 18 years was associated with aBMD in early adulthood, especially in boys. Cross-sectional analyses showed a positive association between moderate physical activity and aBMD.

**Introduction:**

To evaluate independent associations of moderate and vigorous physical activity (MPA, VPA) across adolescence with areal bone mineral density (aBMD).

**Methods:**

Physical activity (PA) was assessed at 11, 15, and 18 years of age by self-report and at 18 years by accelerometry in the 1993 Pelotas Birth Cohort Study. Time spent in MPA and VPA was determined using metabolic equivalents and specific cutoffs based on raw acceleration. Lumbar spine and femoral neck aBMD were measured by DXA at 18 years. Statistical analyses evaluated the association of MPA and VPA with aBMD, after adjusting for skin color, asset index, current height and age at menarche, and peak strain score (based on ground reaction forces of PA).

**Results:**

Lumbar spine and femoral neck aBMD were available for 3947 (49.9% of boys) and 3960 (49.6% of boys) individuals, respectively. Time spent in MPA at 11 and 15 years was not associated with aBMD. VPA at all time points was positively related to both lumbar spine and femoral neck aBMD in boys. Results were consistent for objectively measured VPA. Girls who achieved 75+ minutes/week of VPA in at least two follow-ups showed higher aBMD at 18 years of age. Boys who reached 75+ minutes/week of VPA at all follow-ups had on average 0.117 g/cm^2^ (95% CI: 0.090; 0.144) higher femoral neck aBMD than those who never achieved this threshold.

**Conclusions:**

Self-reported VPA but not MPA throughout adolescence was associated with aBMD. Recommendation for PA in young people should consider the importance of VPA.

## Introduction

Physical activity is important for preventing major non-communicable diseases [1]. It also has a significant role in the development of the peak bone mass during the first decades of life [2]—relevant to prevent osteoporotic fractures in later life. The association between physical activity and bone mass accrual is mainly elucidated by its mechanical effect because bone strength is enhanced in response to increased mechanical loading [3]. Results from previous studies suggested greater magnitude of associations between physical activity and aBMD when activities including high ground reaction forces are considered in the construction of physical activity variables [4–6].

A previous study performed with the 1993 Pelotas Birth Cohort observed similar magnitude of the association between physical activity and bone density according to inclusion of peak strain score, as proposed by Groothausen [7], though coefficients for variables constructed with peak strain score were slightly higher [8]. This draws attention to other mechanisms involved in such association that could be related to other characteristics of physical activity, such as its intensity.

Physical activity has several additional physiological effects related to increase on aBMD, such as decrease in proinflammatory cytokines [9] and increase in substances such as nitric oxide and insulin-like growth factor 1 (IGF-1) [10, 11]. Vigorous-intensity physical activity is particularly important [12–15] to decrease osteoclastic activation and increase osteoblastic activation [3, 16, 17]. Thus, beyond mechanical stress related to impact on bones promoted by physical activity, other physiological aspects related to the intensity of physical activity may be important in bone mass accrual. Accordingly, the intensity of physical activity may be as important as its impact in the association with bone mass—this is not highlighted in current guidelines [18].

Thus, this study aimed at assessing the association between physical activity accumulated during adolescence and areal bone mineral density (aBMD) at age 18, with focus in the independent associations of moderate- and vigorous-intensity physical activities.

## Methods

This study was carried out with participants belonging to the 1993 Pelotas (Brazil) Birth Cohort. Pelotas is a southern Brazilian city with ~ 330,000 inhabitants. In 1993, all maternity hospitals were visited daily and all live-born whose mothers lived in the urban area of the city (*N* = 5249—only 16 refusals) were included in the cohort. This study used information from three follow-up visits of the cohort, when participants were 11, 15, and 18 years of age—these follow-ups aimed to locate all participants of the cohort. All phases of the 1993 Pelotas Birth Cohort Study obtained approval by the Ethics Committee in Research of the Faculty of Medicine at Federal University of Pelotas and written informed consent was obtained from parents or guardians or from participants themselves (in the last follow-up). More details on the methodology of these studies have been published elsewhere [19–21].

We used several strategies to locate the participants (household visits based on the most recent address reported, information obtained during a school census, hospital records, city census, etc.). After locating study participants, at 11 and 15 years, a household visit was performed by trained interviewers using a standardized and pre-tested questionnaire containing questions about several health-related topics. At 15 years, individuals were also invited to visit the Epidemiologic Research Center to perform anthropometric measurements and spirometry, and to donate a blood sample. At 18 years, participants also visited the Epidemiologic Research Center for clinical exams and an extensive lifestyle interview.

Areal bone mineral density—aBMD—(g/cm^2^) was measured at 18 years of age at each femoral neck and at the lumbar spine (L1–L4) by dual-energy x-ray absorptiometry (DXA) (Lunar Prodigy Advance, GE, Germany). Pregnant or suspected pregnant girls, subjects weighing more than 120 kg, or those with metal plates/screws inside the body or metal pieces (piercings, rings, or bracelets) that could not be removed were excluded of DXA scan. The DXA machine was calibrated daily before each session according to manufacturer recommendation. Concerning the information of femoral neck aBMD, we analyzed the average of both right and left sides.

At 11- and 15-year follow-ups, physical activity was measured using a list of activities, making inferences about the type of physical activity, besides the time spent in each activity and the way adolescents travel to/from school [22]. The instrument used at 11 years included the following activities: outdoor soccer, indoor football, athletics, basketball, dance, gymnastics, martial arts, swimming, volleyball, tennis, handball, trapper, and playing bat. Participants also had the opportunity to report additional activities not listed. At 15 years, handball, trapper, and playing bat were removed from the list, whereas we included walking, weight lifting, and fitness training. At 15 years, handball, trapper, and playing bat were replaced with walking, weight lifting, and fitness training. At the 18-year follow-up, adolescents were interviewed using the leisure-time and commuting sections from the International Physical Activity Questionnaire (IPAQ—long version)[23]. Physical activity at each time point was estimated by multiplying frequency by duration for both leisure-time and commuting domains.

Activities reported at 11 and 15 years provided metabolic equivalents (METs) based on the compendium of physical activity for adolescents, proposed by Ridley, et al. [24]. As the compendium of physical activity for adolescents describes three different MET alternatives for each modality, the average of these was used when estimating METs for each activity. Weight lifting would be classified as light intensity activity according to the protocol used and with the compendium. However, in this case, MET was attributable according to the compendium for adults [25]. We used the same procedure for the classification of transport activities. At 18 years, physical activities were classified in moderate or vigorous intensities according to the IPAQ guide for processing and analyzing data (www.ipaq.ki.se). Thereafter, leisure-time and commuting activities were classified as moderate and vigorous intensity (< 3.0 METs = light; 3.0 a 6.0 METs = moderate e > 6.0 METs = vigorous) as proposed by Pate et al. [26].

After classifying the activities according to intensity, two variables related to weekly time of moderate- and vigorous-intensity physical activity for each follow-up were generated: (i) tertiles of moderate and vigorous PA at 11, 15, and 18 years; (ii) moderate and vigorous PA trajectories, based on the following cutoff points: ≥ 150 and ≥ 75 min per week of moderate or vigorous PA practice, respectively. Such thresholds to moderate (150 min/w) and vigorous (75 min/w) intensity physical activity were defined based on a minimal energy expenditure of 13 MET-hours per week which is equivalent to walking 150 min/week at a 4-mile/hour pace or 75 min/week at a 6-mile/hour pace, respectively, as shown in a previous publication [27]. A four-category variable was created according to classification based on the thresholds above, at each specific time point: never, once, twice, and always active.

To investigate if the association of physical activity intensity with aBMD is independent of the impact, physical activity at 11 and 15 years of age was also assessed using the peak strain score purposed by Groothausen [7]. Physical activities with ground reaction forces of less than one time the body weight such as cycling and swimming have a peak score of 0; activities with peak score between one and two times the body weight—weight-bearing activities such as jogging, walking, and dancing—have a peak score of 1; activities with ground reaction forces between two and four times the body weight—including sprinting and turning actions such as tennis, aerobics, and soccer have a peak score of 2; activities including jumping actions with ground reaction forces greater than four times the body weight such as basketball and gymnastics have the peak score 3. The peak scores of each activity were summed independently at each age.

At 18 years old, physical activity was also measured objectively using the accelerometer (GENEActiv ActivInsight, Kimbolton, UK). This device registers physical activity in three axes and expresses the measure in gravity acceleration (1000 mg = 1 g = 9.81 m/s^2^). Adolescents were asked to wear the device in the non-dominant wrist, during all day and night, including when having a shower and performing other water activities (e.g., swimming, washing the dishes). The period of use varied from 4 to 7 days, including one weekend day. Data recording was based in a 5-s “epoch” and a sample frequency (85.7 Hz).

Data were processed in GENEActiv software and analyzed using the R-package GGRI (https://cran.r-project.org/web/packages/GGIR/vignettes/GGIR.html#citing-ggir).

Procedures in the accelerometry analyses were made according to previous publications [28, 29]. Detailed signal processing included: verification of sensor calibration error (using local gravity as reference), detection of sustained abnormally high values, non-wear detection, exclusion of the first 10 and last 20 h of the measurement. Data was imputed for periods with invalid data and the average of similar time points on different days of the measurement was used. Valid data were present for every 15-min period in a 24-h cycle. Time (minutes/day) spent in moderate and vigorous physical activities were calculated in 5-min bouts using thresholds of 100 mg and 400 mg, respectively [30].

Other variables considered as possible confounders were: sex, skin color, age at menarche, and household assets index at 11 years (based on the ownership of self-reported household goods—obtained through factor analysis [31]. Standing height at 18 years was measured to the nearest 1 mm with barefooted subjects using a wooden stadiometer.

Statistical analyses were performed with Stata 12 software (StataCorp, College Station, TX, USA). The analyses were stratified by sex since potential effect modification was considered when the *p* value for the interaction term was 0.2. We used Student’s *t* test to compare the lumbar spine and femoral neck BMD between boys and girls. The linear relationship between time spent in moderate and vigorous physical activity and peak strain score was investigated using Spearman’s rank correlation coefficient. Moderate and vigorous intensity physical activity at each follow-up was stratified into tertiles in all analyses. The relationship between our variables in their continuous form is not linear and the use of tertiles allows observation of a dose-response relationship between our exposures and outcomes. We also evaluated the association of the accumulation of self-reported moderate and vigorous intensity physical activity with aBMD. Estimates were adjusted for sex, skin color, age at menarche, household assets index, height (because body size is highly correlated to bone mass), and also for peak strain score using linear regressions. The significance level was set at 5%.

## Results

A total of 4106 adolescents from the 1993 Pelotas Birth Cohort were interviewed at 18 years (follow-up rate: 81.4%, including the 169 known deaths). DXA scans were obtained in 3947 (49.9% of boys) and 3960 (49.6% of boys) individuals for lumbar spine and femoral neck aBMD, respectively (96.1% and 96.4% from the total interviewed). Coefficient of variation for lumbar spine aBMD was 11.0% in boys and 11.2% in girls and for femoral neck aBMD it was 13.4% and 12.6% in boys and girls, respectively.

Table 1 shows characteristics of the adolescents, stratified by sex. Around 64% of the adolescents were whites and girls were more likely to achieve at least 150 min/week in moderate-intensity physical activity at the first two follow-ups during the adolescence. On the other hand, around half of the boys achieved ≥ 75 min/week in vigorous intensity physical activity at each time point whereas prevalence of girls who reached this cutoff point ranged from 8.5% at 15 years to 20.8% at 18 years of age. Peak strain score was higher at age 11 than at 15 years and higher among boys than in girls. Lumbar spine and femoral neck aBMD was statistically higher in boys and girls.

**Table 1:** Characteristics from adolescents belonging to the 1993 Pelotas Birth Cohort with DXA scans at 18 years of age

	Boys		Girls	
	*N*	%	*N*	%
Skin color				
White	1219	64.6	1307	63.8
Black	278	14.7	290	14.2
Brown	314	16.7	383	18.7
Yellow/indigenous	75	4.0	68	3.3
≥ 150 min/week of moderate PA				
11 years	1038	48.7	1259	57.7
15 years	860	40.7	1145	51.8
18 years (self-reported)	1517	75.3	1327	63.5
≥ 75 min/week of vigorous PA				
11 years	1015	47.7	278	12.7
15 years	1098	51.9	188	8.5
18 years (self-reported)	990	49.3	434	20.8
Peak strain score at 11 years [mean (SD)]	1856	4.09 (3.24)	1895	3.26 (3.15)
Peak strain score at 15 years [mean (SD)]	1862	3.01 (2.37)	1961	2.00 (2.34)
LSaBMD at 18 years (g/cm^2^) [mean (SD)]	1969	1.18 (0.13)	1978	1.16 (0.13)
FNaBMD at 18 years (g/cm^2^) [mean (SD)]	1965	1.19 (0.16)	1995	1.03 (0.13)

*PA* physical activity, *LSaBMD* lumbar spine areal bone mineral density, *FNaBMD* femoral neck areal bone mineral density

Correlation coefficients between self-reported time spent in moderate or vigorous intensity physical activity and peak strain score at 11 and 15 years of age are shown in Table 2. At 11 years, moderate or vigorous physical activity showed similar correlation coefficients with peak strain score. However, at 15 years of age, the correlation coefficient between time spent in vigorous physical activity and peak strain score was higher (rho = 0.57) than that for moderate intensity physical activity (rho = 0.34) in boys, whereas in girls the correlation coefficients between both intensities and peak strain score were similar.

**Table 2:** Spearman’s correlation coefficients between peak strain score and time spent in moderate- and vigorous-intensity physical activity at 11 and 15 years of age in adolescents belonging to the 1993 Pelotas Birth Cohort, stratified by sex

	Moderate physical activity	Vigorous physical activity
11 years		
Boys	0.49 *p* < 0.001	0.55 *p* < 0.001
Girls	0.55 *p* < 0.001	0.60 *p* < 0.001
15 years		
Boys	0.34 *p* < 0.001	0.57 *p* < 0.001
Girls	0.53 *p* < 0.001	0.52 *p* < 0.001

Tables 3 and 4 show the association of moderate and vigorous intensity physical activity at each time point with aBMD at 18 years of age in boys and girls. Moderate intensity physical activity was not associated with lumbar spine and femoral neck aBMD in both sexes after adjustment for potential confounders and peak strain score. The only exception was moderate physical activity at 18 years in association with femoral neck aBMD. Results were consistent for the association between objectively measured moderate-intensity physical activity and aBMD. On the other hand, time spent in vigorous physical activity was significantly associated with both femoral neck and lumbar spine aBMD at all time points independent in a dose-response manner. In girls, vigorous intensity physical activity at 15 years was associated with higher femoral neck aBMD independent of peak strain score. Similarly, self-reported and accelerometry-measured moderate intensity physical activity was positively associated with aBMD at femoral neck in girls at age 18 years. Finally, self-reported, but not objectively measured vigorous-intensity physical activity was associated with femoral neck aBMD in girls at age 18 years. The magnitude of associations was consistently stronger in boys compared with girls.

**Table 3:** Adjusted association between moderate and vigorous physical activity during adolescence and areal bone mineral density at lumbar spine and femoral neck at 18 years in boys from the 1993 Pelotas (Brazil) Birth Cohort

	Areal bone mineral density (g/cm^2^)					
	Lumbar spine (L1–L4)			Femoral neck		
	*n*	β coefficient (95% CI)	*p*	*n*	β coefficient (95% CI)	*p*
Weekly time in MPA at 11 years^a^ (tertiles) [mean (sd)]			0.835			0.286
1st [49.9 (34.9)]	755	Ref.		755	Ref.	
2nd [162.5 (35.1)]	567	0.00 (− 0.02; 0.01)		568	− 0.01 (− 0.03; 0.00)	
3rd [536.8 (432.0)]	528	0.00 (− 0.01; 0.02)		525	− 0.01 (− 0.03; 0.01)	
Weekly time in VPA at 11 years^a^ (tertiles) [mean (sd)]			0.007			< 0.001
1st [0 (0)]	432	Ref.		430	Ref.	
2nd [73.8 (36.3)]	501	0.02 (0.00; 0.04)		500	0.02 (0.00; 0.04)	
3rd [454.5 (358.5)]	915	0.03 (0.01; 0.05)		914	0.04 (0.02; 0.07)	
Weekly time in MPA at 15 years^b^ (tertiles) [mean (sd)]			0.735			0.237
1st [20.2 (22.0)]	704	Ref.		704	Ref.	
2nd [121.9 (34.8)]	634	0.00 (− 0.02; 0.01)		632	− 0.01 (− 0.03; 0.01)	
3rd [483.6 (401.0)]	524	− 0.01 (− 0.02; 0.01)		522	− 0.02 (− 0.03; 0.00)	
Weekly time in VPA at 15 years^b^ (tertiles) [mean (sd)]			< 0.001			< 0.001
1st [0 (0)]	455	Ref.		453	Ref.	
2nd [77.9 (36.0)]	404	0.01 (− 0.01; 0.02)		404	0.03 (0.01; 0.05)	
3rd [540.1 (418.4)]	1003	0.03 (0.02; 0.05)		1001	0.06 (0.04; 0.08)	
Weekly time in MPA at 18 years (tertiles) [mean (sd)]			0.345			0.001
1st [75.5 (51.8)]	525	Ref.		523	Ref.	
2nd [265.3 (67.9)]	663	0.01 (0.00; 0.03)		660	0.02 (0.00; 0.04)	
3rd [980.3 (813.0)]	781	0.01 (− 0.01; 0.02)		782	0.03 (0.02; 0.05)	
Weekly time in VPA at 18 years (tertiles) [mean (sd)]			< 0.001			< 0.001
1st [0 (0)]	822	Ref.		819	Ref.	
2nd [58.6 (17.2)]	216	0.03 (0.01; 0.05)		215	0.03 (0.01; 0.06)	
3rd [378.2 (293.5)]	929	0.04 (0.03; 0.06)		929	0.05 (0.04; 0.07)	
Daily time in objectively measured MPA at 18 years (tertiles) [mean (sd)]			0.181			0.001
1st [94.1 (25.3)]	580	Ref.		578	Ref.	
2nd [157.8 (17.0)]	580	0.01 (0.00; 0.03)		580	0.03 (0.01; 0.05)	
3rd [241.0 (44.8)]	584	0.01 (0.00; 0.03)		583	0.03 (0.01; 0.05)	
Daily time in objectively measured VPA at 18 years (tertiles) [mean (sd)]			< 0.001			< 0.001
1st [4.4 (2.1)]	579	Ref.		576	Ref.	
2nd [12.3 (2.6)]	583	0.03 (0.01; 0.04)		583	0.04 (0.02; 0.06)	
3rd [29.6 (12.7)]	582	0.04 (0.02; 0.05)		582	0.06 (0.05; 0.08)	

*MPA* moderate physical activity, *VPA* vigorous physical activity

Adjusted for skin color, asset index at 11 years, current height and peak strain score at 11^a^ and 15^b^ years of age.

**Table 4:** Adjusted association between moderate and vigorous physical activities during adolescence and areal bone mineral density at lumbar spine and femoral neck at 18 years in girls from the 1993 Pelotas (Brazil) Birth Cohort

	Areal bone mineral density (g/cm^2^)					
	Lumbar spine (L1–L4)			Femoral neck		
	*n*	β coefficient (95% CI)	*p*	*n*	β coefficient (95% CI)	*p*
Weekly time in MPA at 11 years^a^ (tertiles) [mean (sd)]			0.773			0.534
1st [56.6 (32.6)]	599	Ref.		608	Ref.	
2nd [164.0 (36.0)]	585	0.00 (− 0.01; 0.02)		588	0.00 (− 0.02; 0.01)	
3rd [529.2 (392.3)]	688	0.01 (− 0.01; 0.02)		691	0.01 (− 0.01; 0.02)	
Weekly time in VPA at 11 years^a^ (tertiles) [mean (sd)]			0.413			0.572
1st [0 (0)]	1258	Ref.		1270	Ref.	
2nd [60.4 (35.9)]	369	0.01 (− 0.01; 0.03)		372	0.01 (− 0.01; 0.03)	
3rd [352.5 (299.0)]	246	0.01 (− 0.01; 0.03)		246	0.01 (− 0.01; 0.03)	
Weekly time in MPA at 15 years^b^ (tertiles) [mean (sd)]			0.292			0.347
1st [19.0 (21.1)]	559	Ref.		566	Ref.	
2nd [126.5 (35.5)]	642	0.00 (− 0.01; 0.01)		645	− 0.01 (− 0.02; 0.01)	
3rd [451.4 (313.4)]	741	0.01 (0.00; 0.03)		747	0.00 (− 0.01; 0.02)	
Weekly time in VPA at 15 years^b^ (tertiles) [mean (sd)]			0.301			0.035
1st [0 (0)]	1559	Ref.		1570	Ref.	
2nd [65.0 (38.0)]	225	0.01 (− 0.01; 0.03)		227	0.02 (0.00; 0.04)	
3rd [365.1 (339.2)]	158	0.02 (0.00; 0.04)		161	0.03 (0.01; 0.05)	
Weekly time in MPA at 18 years (tertiles) [mean (sd)]			0.062			0.045
1st [70.6 (51.6)]	790	Ref.		797	Ref.	
2nd [255.3 (67.7)]	671	0.00 (-0.01; 0.02)		672	0.01 (0.00; 0.02)	
3rd [825.0 (649.2)]	517	0.02 (0.00; 0.03)		526	0.02 (0.00; 0.03)	
Weekly time in VPA at 18 y (tertiles) [mean (sd)]			0.256			< 0.001
1st [0 (0)]	1478	Ref.		1489	Ref.	
2nd [58.3 (21.4)]	102	0.00 (− 0.02; 0.03)		105	0.00 (− 0.03; 0.02)	
3rd [306.6 (203.6)]	398	0.01 (0.00; 0.03)		401	0.03 (0.01; 0.04)	
Daily time in objectively measured MPA at 18 years (tertiles) [mean (sd)]			0.020			< 0.001
1st [84.5 (19.1)]	593	Ref.		601	Ref.	
2nd [128.8 (11.5)]	604	0.00 (− 0.02; 0.01)		606	0.01 (− 0.01; 0.02)	
3rd [191.6 (40.7)]	601	0.02 (0.00; 0.03)		605	0.03 (0.02; 0.05)	
Daily time in objectively measured VPA at 18 years (tertiles) [mean (sd)]			0.089			0.150
1st [2.0 (1.0)]	597	Ref.		602	Ref.	
2nd [5.7 (1.2)]	599	0.00 (− 0.01; 0.02)		603	0.00 (− 0.01; 0.02)	
3rd [15.7 (8.4)]	602	0.02 (0.00; 0.03)		607	0.01 (0.00; 0.03)	

*MPA* moderate physical activity, *VPA* vigorous physical activity

Adjusted for skin color, asset index at 11 years, current height and peak strain score at 11^a^ and 15^b^ years of age.

**Fig. 1:**
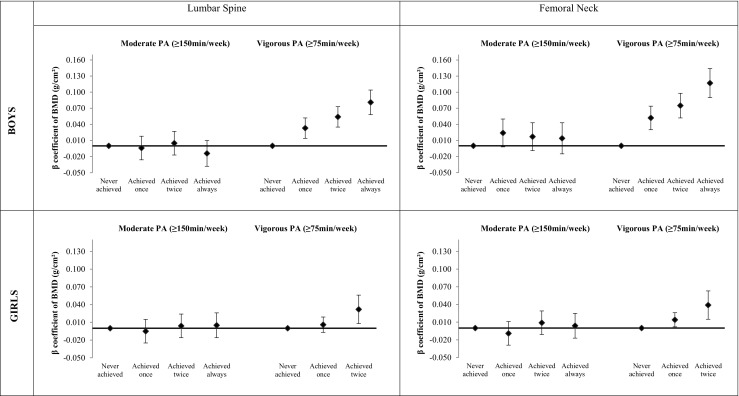
Trajectory of physical activity from 11 to 18 years and areal bone mineral density at lumbar spine and femoral neck in boys and girls belonging to the 1993 Pelotas Birth Cohort

The accumulation at the three follow-ups of time spent in moderate intensity physical activity ≥ 150 min/week was not associated with lumbar spine and femoral neck aBMD in both boys and girls after adjustment for confounders and peak strain score at 11 and 15 years of age. A dose-response association was found between time spent in vigorous physical activity ≥ 75 min/week and aBMD in boys since both lumbar spine and femoral neck aBMD were higher by increasing the number of periods in which the adolescents achieved the cutoff point. Boys who achieved ≥ 75 min/week in vigorous intensity physical activity at all three time points had on average 0.117 g/cm^2^ higher femoral neck aBMD than boys who never achieved this activity level. Girls who reached at least 75 min/week in vigorous physical activity at two follow-ups showed higher aBMD at 18 years of age than girls who never achieved this activity level. Less than 1% of girls were active according to this level at all three time points precluding any robust inference about this category (Fig. 1).

## Discussion

This study investigated longitudinal and cross-sectional associations between physical activity and aBMD with focus in differences according to physical activity intensity. We observed that vigorous physical activity was more strongly related to aBMD than moderate physical activity in both sexes. Results were more consistent in boys, but engagement in vigorous activities from the middle to the end of adolescence seems to be related to higher values of femoral neck aBMD, an important site related to osteoporotic fractures in later life. Although there was moderate correlation coefficient between vigorous physical activity and peak strain score, the association of vigorous-intensity physical activity with aBMD was independent of the impact of physical activity. In general, results were consistent when objectively measured moderate and vigorous physical activities were used in the analyses. Analyses considering the accumulation at the three follow-ups highlighted the results since aBMD at 18 years of age was higher according to the number of follow-ups in which boys reached at least 75 min/week in vigorous physical activity.

Bone density measurements cannot identify individuals who will have a fracture but they predict risk of fracture. A recent meta-analysis showed that − 1 SD in spine aBMD is related to 2.3 times higher risk for vertebral fractures whereas − 1 SD in hip aBMD increases the risk for hip fractures 2.6 times [30]. Boys who did not reach 75 min/week of vigorous physical activity at any time point had a lumbar spine aBMD 0.3 SD below the average of all boys, whereas boys who reached the same criterion at all the three time points had a lumbar spine aBMD 0.3 SD above average. Thus, if these differences in activity levels between groups persist over time, those categorized as active throughout the study period may have around 80% lower risk for vertebral fractures in the future compared to those who did not reach our criterion at any time point. A similar calculation suggests that boys who reached 75 min/week in vigorous-intensity physical activity at every time point have around 120% lower risk for hip fractures than boys who did not reach it. In girls, those in the highest tertile in the time spent in vigorous physical activity at 18 years of age had 40% lower risk for hip fracture than girls in the lowest tertile—accepting the maintenance of the distribution of data over time.

Vigorous physical activity at 11 years of age was also positively related to femoral neck aBMD in girls, prior to the adjustment to peak strain score but adjusted to other confounders in the same age (data not shown). This may indicate long-term importance of vigorous physical activity to bone health that is also dependent of impact of the activities. On the other hand, the magnitude of association between PA and aBMD was positively associated with the intensity of activity and was slightly attenuated after adjustment for peak strain score. This may reinforce the relevance of including the role of vigorous intensity PA for bone health in the public health guidelines [18].

The importance of vigorous intensity physical activity for improving bone mass in our population is strengthened by findings from accumulation or stability in physical activity according to the intensity. Those who achieved the PA guidelines for VPA at all three time points had significantly higher aBMD at age 18 years, however, this was not observed concerning moderate intensity physical activity. This suggests consistency in our results since different instruments were used in these participants and direction and magnitude did not change.

Previous studies have found a sustained effect of physical activity at earlier ages on bone mass during adolescence or young adulthood [32–34]. These findings corroborate with our results concerning the cumulative effect of vigorous physical activity during adolescence on aBMD, indicating that any point of adolescence is a “window of opportunity” to increase bone mass by participating in vigorous-intensity physical activity.

Findings from the current study indicated that the coefficients of the association between vigorous physical activity and aBMD were higher in boys. First, girls spend less time in vigorous physical activity than boys (Median, 11 years–0 vs. 120; 15 years, 0 vs. 150; 18 years, 210 vs. 300 from self-report and 5.6 vs. 12.1). Thus, boys and girls in the same tertile are not exposed to the same amount of time spent in vigorous intensity physical activity. Differences in physical activity between boys and girls and different associations with aBMD were previously described in other cohort studies [35] and corroborate with suggestion of a sensitivity of bone from physical activity that favors males [36, 37]. In addition to higher sensitivity to mechanical loading, boys during adolescence are more exposed to testosterone, an important sexual hormone that mediates several mechanisms engaged on increase of bone mass [38] and muscle mass—which is positively related to bone mass [39]. Taken together, this may explain the greater magnitude of associations from vigorous intensity physical activity on bone density observed in boys.

The use of different instruments to assess physical activity is a limitation of this study. However, results from these different questionnaires were in the same direction, what reinforce our findings. In addition, findings from accelerometers were, in general, consistent with that from self-reported instruments. Unfortunately, we did not have information about types of activities performed at age 18 years precluding the possibility to adjust for peak strain score at this age. On the other hand, we were able to discriminate between objectively measured moderate and vigorous intensity physical activity. Finally, repeated information on physical activity from the beginning to the end of adolescence made it possible to examine associations at each time point individually and in a combined physical activity score, although group-based trajectory models could not be described due to the characteristics of the data distribution. Another important limitation is that we do not have information on aBMD at previous time points. Thus, we could not adjust the analyses for previous aBMD. Ultimately, we do not have information on ethnicity. However, as reported in the 1982 Pelotas Cohort, self-classification as black and white is strongly correlated with individual proportions of African and European ancestry [40].

Because we tested for the association of eight different exposures with two different outcomes, the observed associations might have occurred by chance due to inflations in type-1 error. However, it is important to consider that these exposures, as well as both outcomes, are correlated with one another, so the inflation is smaller than one would expect based on the number of exposures alone. Moreover, the association for moderate and vigorous physical activity at 18 years with lumbar spine and femoral neck aBMD was observed also considering objectively measurement of physical activity and the observed number of associations that achieved conventional levels of statistical significance was higher than what would be expected by chance alone.

In summary, we conclude that vigorous-intensity physical activity was associated with higher bone density at both lumbar spine and femoral neck, relevant anatomical sites of osteoporotic fractures, during adolescence—a critical time of peak bone gain—whereas time spent in moderate physical activity was not associated with bone mass. Results were independent of impact of the activities and suggested that physical activity guidelines should take into account the importance of vigorous intensity physical activity to improve bone health. The findings were more pronounced in boys than in girls. However, based on our results, vigorous intensity physical activity probably has potential to avoid future health problems related to low bone density in both sexes.
